# Early Fungicidal Activity as a Candidate Surrogate Endpoint for All-Cause Mortality in Cryptococcal Meningitis: A Systematic Review of the Evidence

**DOI:** 10.1371/journal.pone.0159727

**Published:** 2016-08-04

**Authors:** Jairo M. Montezuma-Rusca, John H. Powers, Dean Follmann, Jing Wang, Brigit Sullivan, Peter R. Williamson

**Affiliations:** 1 Laboratory of Immunoregulation, National Institute of Allergy and Infectious Diseases, National Institutes of Health, Bethesda, MD, United States of America; 2 Clinical Research Directorate, Clinical Monitoring Research Program, Leidos Biomedical Research, Inc., Frederick National Laboratory for Cancer Research, Frederick, MD, United States of America; 3 Mathematical Statisticians, Biostatistics Research Branch, National Institute of Allergy and Infectious Diseases, National Institutes of Health, Bethesda, MD, United States of America; 4 National Institutes of Health Library, Office of Research Services, National Institutes of Health, Bethesda, MD, United States of America; 5 Laboratory of Clinical Infectious Disease, National Institute of Allergy and Infectious Diseases, National Institutes of Health, Bethesda, MD, United States of America; Rutgers University, UNITED STATES

## Abstract

**Background:**

Cryptococcal meningitis (CM) is a leading cause of HIV-associated mortality. In clinical trials evaluating treatments for CM, biomarkers of early fungicidal activity (EFA) in cerebrospinal fluid (CSF) have been proposed as candidate surrogate endpoints for all- cause mortality (ACM). However, there has been no systematic evaluation of the group-level or trial-level evidence for EFA as a candidate surrogate endpoint for ACM.

**Methods:**

We conducted a systematic review of randomized trials in treatment of CM to evaluate available evidence for EFA measured as culture negativity at 2 weeks/10 weeks and slope of EFA as candidate surrogate endpoints for ACM. We performed sensitivity analysis on superiority trials and high quality trials as determined by Cochrane measures of trial bias.

**Results:**

Twenty-seven trials including 2854 patients met inclusion criteria. Mean ACM was 15.8% at 2 weeks and 27.0% at 10 weeks with no overall significant difference between test and control groups. There was a statistically significant group-level correlation between average EFA and ACM at 10 weeks but not at 2 weeks. There was also no statistically significant group-level correlation between CFU culture negativity at 2weeks/10weeks or average EFA slope at 10 weeks. A statistically significant trial-level correlation was identified between EFA slope and ACM at 2 weeks, but is likely misleading, as there was no treatment effect on ACM.

**Conclusions:**

Mortality remains high in short time periods in CM clinical trials. Using published data and Institute of Medicine criteria, evidence for use of EFA as a surrogate endpoint for ACM is insufficient and could provide misleading results from clinical trials. ACM should be used as a primary endpoint evaluating treatments for cryptococcal meningitis.

## Introduction

Cryptococcal meningitis (CM) is a neglected disease, although it is a leading cause of HIV/AIDS-associated mortality in sub-Saharan Africa with an estimated half million deaths yearly. [1] Combined antiretroviral therapy (cART) has decreased HIV/AIDS–associated mortality in the developed world such as the United States [2]; however, access to cART is still limited in many regions globally and is often a first presentation of HIV infection. In addition, even after ‘standard of care’ therapy, mortality remains high; thus, effective antifungal therapies are still needed to decrease mortality and disability caused by CM.[3] To facilitate drug development, authors have hypothesized that microbiological biomarkers may serve as candidate trial-level surrogate endpoints replacing all-cause mortality (ACM) in assessing treatment effects of antifungal drugs in CM to decrease the number of enrolled participants in trials.[4] Differences in proportions of cerebrospinal fluid (CSF) cultures below the level of detection (CFU<LOD; referred to here as CSF culture negativity) at 2 and 10 weeks and the difference in mean slope of quantitative cultures based on the number of colony forming units (CFUs) in the CSF over time (EFA) have been the most commonly used measurements of microbiological clearance in patients with CM.[5–12] Indeed, this biomarker was utilized as part of a composite endpoint to recommend trial suspension in a recent trial of adjunctive corticosteroids in treating CM [13]. However, prior to implementation, rigorous analysis of such endpoints will help delineate the advantages and limitations of biomarkers in clinical studies and patient care. Indeed, confusion in the use of a microbial biomarker in the treatment of AIDS-associated mycobacterial disease with clarithromycin may have contributed to excess trial mortality in one recent study [14].

The Institute of Medicine (IOM) framework for evaluation of biomarkers as candidate surrogate endpoints recommends both analytical validation and qualification assessments for biomarker evaluation. Evaluation of analytical validity of biomarkers includes assessments of reliability, reproducibility, standardization and quality controls. Qualification requires two further criteria to consider a biomarker as a valid candidate surrogate endpoint. The biomarker should serve as a group-level correlate with the true direct clinical endpoint (ACM in this disease) regardless of therapy. This requires evaluation of a correlation between quantitative changes in the biomarker with changes in the direct measure of patient benefit independent of treatment (higher or lower concentrations of CFUs in the CSF or higher/lower slope of EFA with ACM regardless of which, or any, treatment received without a test or control group) across multiple studies. The next criteria is that the biomarker should serve as a trial-level surrogate in that a net treatment effect (difference in outcome between the test and control group related to treatment) of interventions on the biomarker should capture the net treatment effect of the intervention on the clinical endpoint [15]. In other words, there should be treatment related changes between the test and control group on both the biomarker and the direct outcome of interest. This is based on an evaluation of *differences* between test and control group on the biomarker (EFA) compared to *differences* in the direct patient outcome (ACM). Implicit in this construction is that the treatment has an effect on *both* the biomarker and the direct patient endpoint.[16] For general applicability, the biomarker should be valid across a number of study conditions with different drugs and different patient populations. The IOM recommends an initial evaluation of a biomarker as a surrogate endpoint should consider all studies in a disease area in a trial-level meta-analysis.[17] [18] Such analyses have been carried out for various surrogate endpoints like progression free survival in oncology and HIV viral load in AIDS.[18] [19,20]

Although EFA has been shown to correlate with ACM independent of treatment in several individual clinical trials (group-level surrogacy) [21], group-level correlations have not been assessed across all trials. Furthermore, while these correlations represent a first step in evaluation of a biomarker they do not assess the relationship between treatment related changes in EFA and treatment effects on ACM (trial-level surrogacy). Further evaluation at the trial level of treatment effects across all trial evidence is needed to recommend the use of EFA as a reliable surrogate endpoint to assess treatment effects on ACM. To date there has been no systematic trial-level analysis of EFA as a candidate surrogate endpoint in CM. Therefore, we evaluated the available evidence for the biomarkers of cryptococcal EFA slope and culture negativity on both criteria for candidate surrogate endpoints: 1) group-level correlations of measures of EFA with ACM independent of treatment across high quality, randomized control trials, and 2) trial-level treatment effects (differences between test and control groups) on the surrogate of EFA analyzed by two different methods (CSF culture negativity at fixed time points and slope of change in CFUs in CSF [EFA slope]) compared to treatment effects on ACM at 2 and 10 weeks in a systematic review of the literature.

## Materials and Methods

## Search strategy

**Fig 1:**
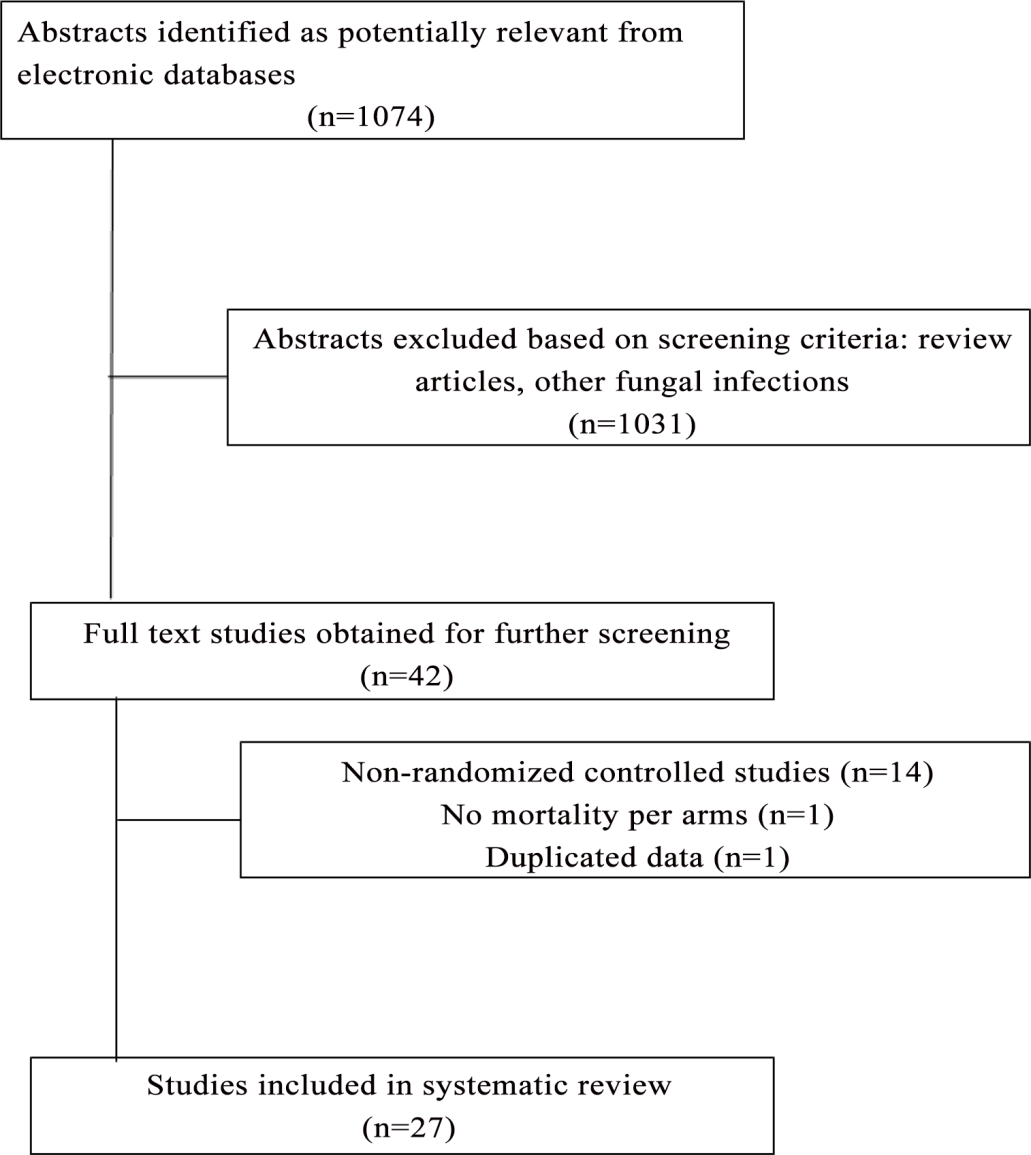
Flow diagram of search strategy and studies selected.

The search sources included Embase, WOS, Scopus, Pubmed, Cochrane Library and Google Scholar through April 22, 2013. The search terms included “cryptococcal meningitis” and “cryptococcosis”. Studies were included if they were randomized trials on treatment of CM. Non-randomized studies, duplicate data, or prevention or maintenance therapy trials for CM were excluded. Microbiological outcomes including average EFA slope and proportions of patients with CSF culture negativity at 2 or 10 weeks and all-cause mortality data at 2 or 10 weeks were extracted (Fig 1).

### Data extraction

Extraction of data was conducted by one investigator and was reviewed by 2 independent investigators. The protocol for the present review was registered in PROSPERO, international prospective register of systematic review from the Centre for Review and Dissemination, University of York (PROSPERO2013:CRD42013003726).

### Risk of bias assessment

To evaluate the quality of the studies we used the Cochrane Collaboration´s tool for assessing risk of bias [22]. Each study was evaluated for 7 types of potential biases. The category of “other bias” included studies with no stated hypotheses and studies with small numbers of patients randomized as studies with less than 20 patients per study arm were considered to have high risk for bias. “Unclear risk” was assigned when information was not available for the respective bias assessment.

Included studies were conducted in the US, Canada, India, Vietnam, Thailand, Indonesia, Laos, Vietnam, Uganda, South Africa, Malawi, Botswana, Zimbabwe and Netherlands.

Antifungal interventions evaluated in the studies were: Amphotericin B deoxycolate (A), liposomal Amphotericin B, lipid complex Amphotericin B, fluconazole (FLU), voriconazole, itraconazole, interferon gamma, rifampin and flucytosine (F) alone or in combination. Early versus delayed cART was evaluated in two studies. One study evaluated the effect of dexamethasone in patients receiving antifungal standard of care. Nineteen studies compared 2 treatment groups, 5 studies compared 3 treatment groups and 3 studies compared 4 treatment groups. (S1 Table)

We defined higher quality studies as those performed in HIV patients in which 3 or more elements were listed as “low risk for bias” and 2 or less elements were listed as “high risk” of bias. The number of “unclear risk” elements in the quality assessment was not considered in evaluating studies for the sensitivity analysis on higher quality studies.

We evaluated correlations of ACM with average EFA slope and proportion of patients with CSF culture negativity at 2 and 10 weeks, and analyses were repeated limited to higher quality studies according to the Cochrane Collaboration´s tool and studies with superiority hypotheses, since trials with non-inferiority hypotheses may minimize differences between test and control groups.

### Statistical Analysis

Descriptive statistics were used to evaluate the results of each treatment group on EFA and ACM. A weighted least squares method with generalized estimating equations (GEE) was used to estimate the relationship between ACM and various measures of EFA. The data from each study arm was weighted by the sample size. Group-level correlation between the mortality rates from treatment groups within the same study was addressed by GEE using study as a cluster variable with a working independence model. The method of Fay and Graubard was used to provide accurate p-values for hypothesis testing.[23]

Analyses to assess trial-level treatment effects were performed in studies comparing control groups usually consisting of fluconazole (FLC, or Amphotericin B (A) regimens alone or in combination with flucytosine (AF) compared to test interventions including various drug combinations, different doses of drugs, lipid formulations of Amphotericin B, interferon, or dexamethasone. (S1 Table)

Between treatment-assignment group contrasts were formed for the test groups compared to the control groups. We tested for trial-level treatment effects on ACM and measures of EFA using weighted least squares as described above with weights proportional to the estimated variance of the contrasts. Study was used as a cluster variable.

We conducted and reported this study using the PRISMA guidelines for systematic reviews.[24]

## Results

### Study characteristics

One-thousand seventy four publications were retrieved from the search, of which 1047 were excluded based on *a priori* exclusion criteria (Fig 1). Twenty-seven studies (n = 2854 patients) were included in this review, encompassing 65 treatment-assignment groups (study arms) and 38 comparisons of test and control groups. Twenty-five studies were in HIV/AIDS patients and 2 studies in non-HIV/AIDS patients; 13 higher quality studies (low bias highlighted in green) were selected for further analysis based on the Cochrane Collaboration tool for assessing risk of bias (Table 1) [22].

**Table 1:** Cochrane´s assessment tool for assessing risk of bias.

Author(s)	Year of publication	Random Sequence generation (Selection bias)	Allocation Concealment (Selection bias)	Blinding of participants and researchers (Performance bias)	Blinding of outcome assessment (Detection bias)	Incomplete outcome data (Attrition bias)	Selective reporting (Reporting bias)	Other bias
**Studies in HIV patients**								
Jarvis et al [5]	2012	**Low**	**Unclear**	**High**	**Unclear**	**Low**	**Low**	**Low**
Techapornroong et al [31]	2007	**Low**	**Low**	**Low**	**Unclear**	**High**	**High**	**High**
Brouwer et al [6]	2004	**Low**	**Unclear**	**High**	**Unclear**	**Low**	**Low**	**High**
Nussbaum et al [7]	2009	**Low**	**Unclear**	**Unclear**	**Unclear**	**Low**	**Low**	**Low**
Mayanja-Kizza et al [32]	1998	**Low**	**Unclear**	**Unclear**	**Unclear**	**Low**	**High**	**High**
Hamill et al [33]	2010	**Low**	**Unclear**	**Low**	**Unclear**	**Low**	**Low**	**Low**
Chotmongkol et al [34]	1997	**High**	**Unclear**	**Unclear**	**Unclear**	**Low**	**Low**	**High**
Tansuphaswadikul et al [35]	2006	**Unclear**	**Unclear**	**Unclear**	**Unclear**	**High**	**High**	**High**
Loyse et al [8]	2012	**Low**	**Unclear**	**Unclear**	**Unclear**	**Low**	**Low**	**High**
Bicanic et al [9]	2008	**Low**	**Unclear**	**High**	**Unclear**	**Low**	**Low**	**Low**
Chotmongkol et al [36]	2005	**Unclear**	**Unclear**	**High**	**Unclear**	**Low**	**Low**	**High**
Jadhav et al [37]	2010	**Low**	**Low**	**Unclear**	**Unclear**	**High**	**High**	**High**
Jackson et al [10]	2012	**Low**	**Unclear**	**Unclear**	**Unclear**	**Low**	**Low**	**High**
Pappas et al [38]	2009	**Unclear**	**Unclear**	**High**	**Unclear**	**Low**	**Low**	**Low**
Pappas et al [39]	2004	**Unclear**	**Unclear**	**Unclear**	**Unclear**	**Low**	**Low**	**High**
Bisson et al [11]	2013	**Unclear**	**Unclear**	**High**	**Unclear**	**Low**	**Low**	**High**
Larsen et al [40]	1990	**Unclear**	**Unclear**	**Unclear**	**Unclear**	**Low**	**Low**	**High**
de Gans et al [41]	1992	**Unclear**	**Unclear**	**Unclear**	**Unclear**	**Low**	**Low**	**High**
Leenders et al [42]	1997	**Low**	**Low**	**Unclear**	**Unclear**	**High**	**High**	**High**
van der Horst et al [43]	1997	**Unclear**	**Unclear**	**Unclear**	**Unclear**	**Low**	**Low**	**Low**
Day et al [44]	2013	**Low**	**Unclear**	**High**	**Unclear**	**Low**	**Low**	**Low**
Makadzange et al [45]	2010	**Low**	**Low**	**High**	**Unclear**	**Low**	**Low**	**Low**
Saag et al [46]	1992	**Low**	**Unclear**	**Unclear**	**Unclear**	**Low**	**Low**	**Low**
Sharkey et al [47]	1996	**Unclear**	**Unclear**	**Unclear**	**Unclear**	**High**	**High**	**High**
Beardsley et al [13]	2016	**Low**	**Low**	**Unclear**	**Unclear**	**Low**	**Low**	**Low**
**Studies including non-HIV patients**								
Bennett et al [48]	1979	**Low**	**Unclear**	**Unclear**	**Unclear**	**Low**	**Low**	**Low**
Dismukes et al [49]	1987	**Unclear**	**Unclear**	**Unclear**	**Unclear**	**High**	**High**	**High**

The mean CD4 count was reported in 19 of 25 studies in HIV/AIDS and ranged from 9 to 100 cells mm^3^. Mean HIV viral load reported in 8 studies ranged from 98,752 to 398,107 copies/ml. Mean age among all studies ranged from 28 to 52 years old.

### Event rates for microbiological and mortality outcomes

**Fig 2:**
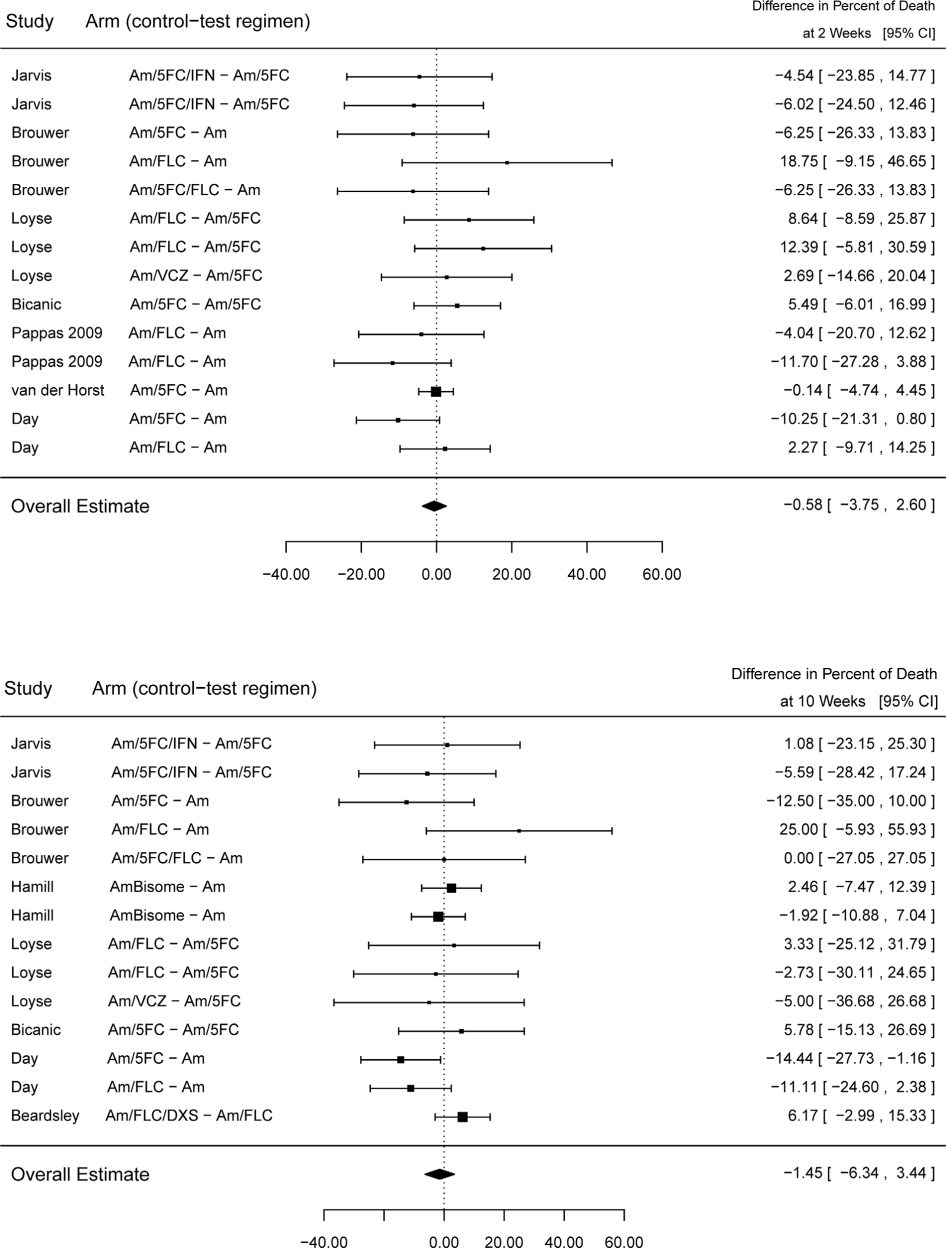
ACM differences between test and control groups at 2 weeks and 10 weeks. All studies with non-missing data are displayed. There are 7 studies and 21 arms.

Not all data was available in every study. Mean ACM was 15.8% and 27.0% at 2 weeks (17 studies, 41 arms) and 10 weeks (17 studies, 40 arms). There was no statistically significant overall treatment effect among trials on ACM with a mortality difference of -0.58% (95% CI -3.75% to +2.60%) at 2 weeks and 1.45% (95% CI -6.34% to +3.44%) at 10 weeks. (Fig 2, top panel)

Mean rates of CSF culture negativity were 42.5% (SD ±0.24) and 63.3% (SD ±0.20) at 2 weeks (11 studies, 27 treatment groups) and 10 weeks (5 studies, 10 treatment groups), respectively. Mean EFA slope was -0.41 cfu/ml CSF/day (SD ±0.13) among studies that measured this microbiological outcome (9 studies, 24 treatment groups).

Among studies that reported CSF culture negativity at 2 weeks, 8 studies reported ACM at 2 weeks, and 5 studies at 10 weeks. Mean EFA slope was reported in 9 studies, of which 8 reported ACM at 2 weeks and 8 reported ACM at 10 weeks.

### Group-level correlations of EFA biomarkers with all-cause mortality independent of treatment

**Fig 3:**
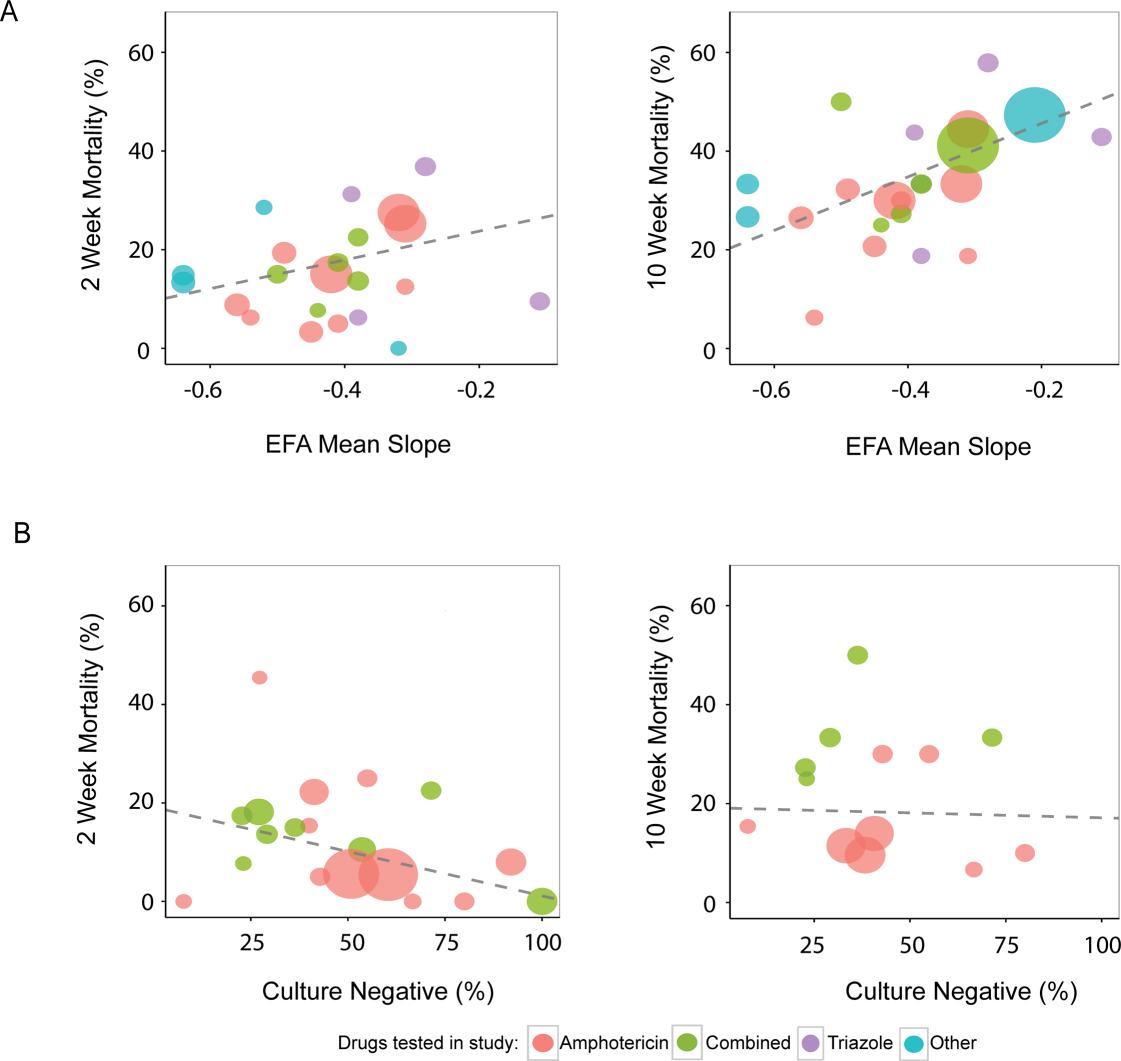
Group-level correlation of EFA and ACM regardless of treatment, all studies. All studies with non-missing data are displayed. N = 16 studies and 40 arms A) Mean EFA slope vs ACM at 2 and 10 weeks (9 studies and 24 arms). B) % CSF culture negative vs. ACM at 2 and 10 weeks (9 studies and 22 arms).

Considering each analysis separately, there was no statistically significant correlation between average EFA slope and ACM at 2 weeks (slope 29.15, 95%CI -24.99 to +83.18, P = 0.18) or between CSF culture negativity at 2 weeks and ACM at 2 weeks (slope -0.18, 95% CI -0.44 to +0.09, P = 0.10) and or 10 weeks (slope -0.02, 95% CI -0.48 to +0.43, P = 0.88). There was a statistically significant correlation between EFA and ACM at 10 weeks (slope 54.2, 95% CI 6.66 to 101.75, P = 0.04) (Fig 3).

Based on the Cochrane assessment of risk for bias, 13 higher quality studies were selected to perform sensitivity analysis. (Table 1) There was no significant correlation between average EFA slope and ACM at 2 weeks (P = 0.14) or between CSF culture negativity at 2 weeks and ACM at 2 or 10 weeks (P = 0.18 and 0.71 respectively). (S1 Fig, S2 Table). A significant correlation between EFA and ACM was again noted at 10 wks (P = 0.04). This relationship was again found significant between EFA and ACM at 10 weeks, considering only trials with superiority hypotheses (P = 0.04). (S2 Fig, S3 Table).

### Trial-level correlation of treatment effects on EFA biomarkers and all-cause mortality

**Fig 4:**
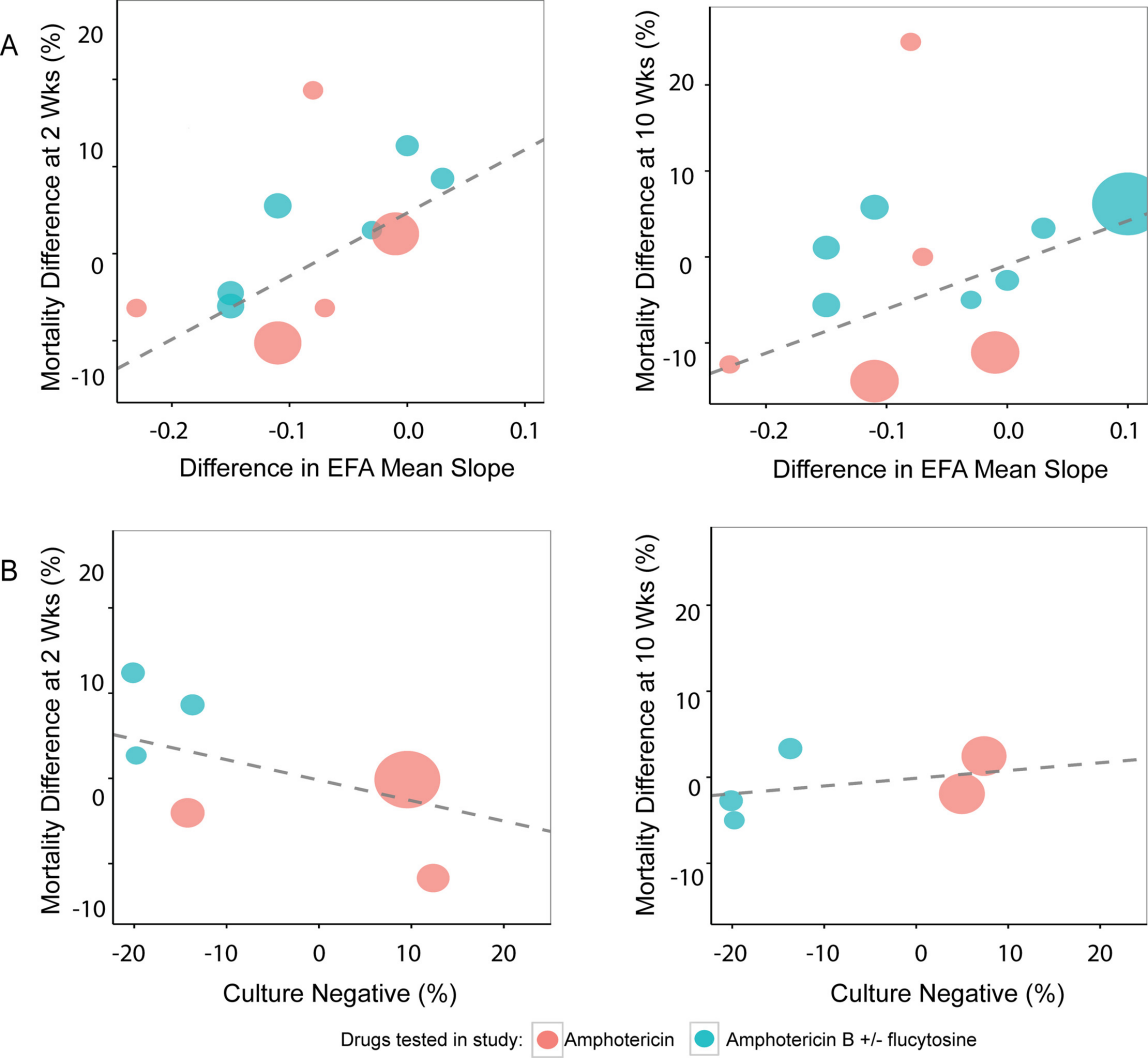
Trial–level correlations of treatment effect on EFA compared to treatment effects on ACM: All studies with non-missing data are displayed. N = 9 studies and 26 arms A) Average EFA slope vs ACM at 2 and 10 weeks (6 studies and 18 arms). B) % CSF culture negative vs ACM at 2 and 10 weeks (4 studies and 12 arms).

In the absence of a consistent significant effect on ACM independent of treatment (Fig 3), the current data do not provide a strong rationale to support testing biomarkers as treatment surrogates. However, since the biomarker is being used as a treatment surrogate and trials have been stopped based in part on this surrogate endpoint [13], we conducted such an analysis. Restricting analyses to high quality studies, there were no statistically significant trial-level correlations between treatment effects on CSF culture negativity at 2 weeks and 2 week ACM (estimate -0.24, 95%CI -0.99 to +0.52, P = 0.23) or 10 week ACM (estimate 0.09, 95% CI -0.03 to +0.22, P = 0.07). There was also no statistically significant correlation between average EFA slope and ACM at 10 weeks (slope estimate 51.31, 95% CI -104.04 to +206.66, P = 0.18). However, there was a statistically significant correlation between EFA slope and ACM at 2 weeks (slope estimate 72.57, 95% CI +11.12 to +134.02, P = 0.03; Fig 4A)

## Discussion

Theoretically, EFA has an important characteristic as a surrogate endpoint in that it is on the causal pathway of disease. Correlation of EFA and mortality has been reported in individual randomized trials, which supports the concept of EFA as a potential candidate surrogate endpoint. According to the IOM, for a biomarker to be a candidate surrogate endpoint it should correlate at the group-level with the direct endpoint regardless of treatment (no comparison of outcomes between test and control groups). If such a correlation is shown, four further criteria are required on trial-level treatment effects: 1) treatment has effect on microbiological outcomes, 2) treatment has an effect on mortality 3) microbiological outcomes have an effect on mortality and 4) the treatment effect on mortality is captured by the treatment effect on microbiological outcomes.[15,17,25] Our analyses utilizing IOM guidelines and published data showed that EFA passes the first of these criteria; however, there was a lack of support for the second criterion (no effect on mortality) and no consistent support for the third criterion (microbiological effects and mortality), making examination of the fourth criterion (treatment effects on microbiological outcomes and mortality) moot. Negative CSF cultures at 2 weeks and 10 weeks also did not meet any of these criteria. Study quality assessed by Cochrane criteria did not affect the outcomes of the analyses as correlation of negative CSF cultures at 2 weeks with ACM at 2 or 10 weeks was not statistically significant in higher quality studies. Evaluation of studies with superiority hypotheses (since non-inferiority hypotheses may minimize differences between groups) also revealed no consistent statistically significant correlation between EFAs or negative CSF cultures with ACM. This lack of correlation shows that EFA does not consistently meet the first criteria and, according to IOM criteria, does not support its role as a candidate treatment surrogate for ACM based on current evidence.

While EFA might present a misleading picture of effects on ACM, because of the significant association at 10 wks and decisions on current trials made based on EFA, we compared trial-level treatment effects on EFA and negative CSF cultures at 2 weeks and 10 weeks, to treatment effects on ACM at 2 and 10 weeks. Our analyses show that treatment again demonstrates inconsistent effects on EFA and ACM, significant (without adjustment for multiple end point comparisons) at 2 weeks but not at 10 weeks in contrast to the group-level analysis. There was no treatment effect on negative CSF cultures at 2 weeks or 10 weeks. Since there is no treatment effect on ACM, the apparent effects on average EFA slope at 2 weeks could present a misleading picture of benefit on the patient-centered outcome of ACM. Thus, one cannot substitute a candidate surrogate endpoint for a direct measure of patient benefit without an effect on the direct endpoint of ACM, according to IOM guidelines. The precision around the estimates of ACM shows that lack of demonstrated differences in ACM is not related to insufficient sample size across all the studies combined.

It is important to note that the present study utilized only randomized controlled trials to facilitate testing for treatment surrogates. Inclusion of lower quality studies such as non-controlled observational studies utilizing clearly inferior therapies such as oral fluconazole that results in a lack of CSF fungal clearance and 100% mortality [26] may improve a group-level prognostic relationship between EFA and ACA but is not valid for evaluating trial-level treatment effects. In addition, a relationship valid principally for therapies largely abandoned (fluconazole monotherapy) is less likely to be useful for identifying therapies superior or equal to current standard-of-care therapies such as intravenous Amphotericin B, nor is applicable as a surrogate endpoint for current trials. Despite its potential correlation within selected patient groups [21], lack of an association of EFA as a group-level prognostic marker across all available studies may also be due to issues with analytical validity reflected in an inability to replicate the EFA assay among different investigators that could hamper its use in clinical practice. Since lack of assay standardization can be a cause of poor correlations, assay validity is the first step in the IOM recommended evaluation. Indeed, we could not find any published literature testing widely utilized reliability measures of EFA testing. We also did not find consistent measurement in trials of other direct patient centered outcomes other than ACM such as patient morbidity to compare to EFA. Mortality is a competing risk for measures of morbidity, as symptoms and patient function cannot be measured in patients who have died; thus, any analysis of morbidity would still need to include mortality. Regarding the analysis of EFA as a treatment surrogate, microbiological biomarkers of treatment efficacy may fail to correlate with direct patient outcomes because of off-target toxicities or benefits, issues with measurement of the biomarker (analytical validity), or alternative mechanisms of disease or treatment benefit not captured by the biomarker.[15,27] While theoretically compelling, microbiological biomarkers do not account for host response to infection, drug toxicities and interactions.[28] The utility of biomarkers of EFA and culture negativity in pre-clinical studies to demonstrate biological activity and prioritize candidates for further development is unclear, as we did not perform analyses to evaluate this as done in oncology.[20] However, the evidence does not support the use of EFA biomarkers as surrogate endpoints for ACM in trials used to confirm patient benefit.

Future studies could be performed to improve the use of EFA as a predictor of individual patient outcomes measured at the patient-level. Patient-level data was not available to perform analyses to evaluate EFA as a prognostic indicator for individual patients in clinical practice. However, trial-level data are necessary to evaluate the use of a biomarker as a treatment surrogate endpoint as we have evaluated here. We found potential biases in many of the randomized trials in CM. Trials in CM often consisted of multiple intervention groups and small numbers of patients enrolled, decreasing the precision of trial results. Poor funding has been implicated as limiting more extensive study of a neglected disease that, nevertheless, kills over a million individuals yearly [29]. More focused trials comparing a smaller number of regimens with an adequate number of patients followed for a sufficient duration of time using ACM as a primary endpoint, and microbiological outcomes as secondary endpoints may be an alternative approach to provide informative surrogate validation. Other direct measures of patient morbidity could be evaluated in addition to ACM, for instance in a ranked ordinal scale [30].

In summary, we utilized published evidence to evaluate EFA biomarkers as candidate surrogate endpoints for ACM in CM trials. Such trial-level meta-analyses have been widely performed to evaluate candidate surrogate endpoints in areas like oncology.[18,20] However, despite widespread use of microbiological biomarkers as surrogate endpoints in infectious diseases trials such as urinary tract infections, streptococcal pharyngitis, gonorrheal urethritis, tuberculosis, and hepatitis C (and others), this is the first trial-level meta-analysis of surrogate endpoints in infectious diseases published in the medical literature outside of HIV.[19] Application of such rigorous analyses of treatment surrogates thus may be important to facilitate the development of anti-infectives to combat the world-wide deficiency of effective antimicrobials.

## Supporting Information

S1 Fig Individual-level Correlations between EFA biomarkers and ACM: High Quality Studies. 1A) EFA mean slope vs ACM at 2 and 10 wks: 8 studies, 22 arms 1B) CSF culture negativity vs. ACM at 2 and 10 wks: 5 studies, 14 arms.
(EPS)

S2 Fig Individual-level Correlations between EFA biomarkers and ACM: Superiority Studies. 1A) EFA mean slope vs ACM at 2 and 10 wks: 7 studies, 16 arms. 1B) CSF culture negativity vs. ACM at 2 and 10 wks: 7 studies, 15 arms.
(EPS)

S1 Table for Fig 3. (DOCX)

S2 Table for Fig 4. (DOCX)

S3 Table Minimal Data Set. (XLSX)

S4 Table PRISMA 2009 checklist. (DOC)
