# Supplementary material for: Early Fungicidal Activity as a Candidate Surrogate Endpoint for All-Cause Mortality in Cryptococcal Meningitis: A Systematic Review of the Evidence
Source: PLoS One. 2016 Aug 4;11(8):e0159727. doi: 10.1371/journal.pone.0159727 (PMC4974008; doi:10.1371/journal.pone.0159727)
Supplement: S1 Table — (DOCX) [file pone.0159727.s003.docx]

**S1 Table for Fig 3:**

| Description | Slope | 95% LCL | 95% UCL | P-value  (SAWS) |
| --- | --- | --- | --- | --- |
| % ACM at 2wks vs. mean slope EFA | 29.15 | -24.88 | 83.18 | 0.18 |
| % ACM at 10 wks vs. mean slope EFA | 54.20 | 6.66 | 101.75 | 0.04 |
| % ACM at 2 wks vs. % CSF culture neg | -0.18 | -0.44 | 0.09 | 0.10 |
| % ACM at 10 wks vs. % CSF culture neg | -0.02 | -0.48 | 0.43 | 0.88 |
