# Supplementary material for: Early Fungicidal Activity as a Candidate Surrogate Endpoint for All-Cause Mortality in Cryptococcal Meningitis: A Systematic Review of the Evidence
Source: PLoS One. 2016 Aug 4;11(8):e0159727. doi: 10.1371/journal.pone.0159727 (PMC4974008; doi:10.1371/journal.pone.0159727)
Supplement: S2 Table — (DOCX) [file pone.0159727.s004.docx]

**S2 Table for Fig 4:**

| Description | Slope | 95% LCL | 95% UCL | P-value  (SAWS) |
| --- | --- | --- | --- | --- |
| Mortality difference at 2wks vs. Difference in mean slope CFUs | 72.57 | 11.12 | 134.02 | 0.03 |
| Mortality difference at 10wks vs. Difference in mean slope CFUs | 51.31 | -104.04 | 206.66 | 0.18 |
| Mortality difference at 2wks vs. Difference in %CSF culture neg | -0.24 | -0.99 | 0.52 | 0.23 |
| Mortality difference at 10wks vs. Difference in %CSF culture neg | 0.09 | -0.03 | 0.22 | 0.07 |
